# Porcine hemagglutinating encephalomyelitis virus nucleocapsid protein targets RIG-I and IRF3 to evade IFN immunity

**DOI:** 10.1128/jvi.02112-25

**Published:** 2026-03-30

**Authors:** Shaoqian Mu, Yuanmao Bai, Ruizhao Qiu, Feilin Zhang, Junchao Shi, Yungang Lan, Feng Gao, Wenqi He, Zi Li

**Affiliations:** 1 State Key Laboratory for Diagnosis and Treatment of Severe Zoonotic Infectious Diseases, Key Laboratory for Zoonosis Research of the Ministry of Education, Institute of Zoonosis, and College of Veterinary Medicine, Jilin University, Changchun, China; Loyola University Chicago - Health Sciences Campus, Maywood, Illinois, USA

**Keywords:** porcine hemagglutinating encephalomyelitis virus, nucleocapsid protein, immune evasion, RIG-I, IRF3

## Abstract

The neurotropic betacoronavirus porcine hemagglutinating encephalomyelitis virus (PHEV) subverts early innate defenses to establish persistent neuronal infection. We show that PHEV activates RIG-I-MAVS signaling but hijacks this pathway to induce a delayed IRF7-dependent interferon (IFN-I) response (>12 h post-infection), permitting unchecked replication prior to late-phase immunity. Mechanistically, the viral nucleocapsid (N) protein directly engages RIG-I’s caspase activation and recruitment domain (CARD) via its C-terminal domain (CTD), competitively blocking TRIM25-mediated K63-linked ubiquitination and silencing RIG-I activation. Concurrently, N protein disrupts IRF3 activation by disrupting homodimerization, phosphorylation, and nuclear translocation, abrogating its function as the dominant early antiviral mediator. Consequently, inadequate IRF7-driven IFN induction (<3-fold at mRNA level) fails to compensate for IRF3 inactivation, creating an immune-permissive window. Pharmacological blockade of replication (Remdesivir or Lopinavir) abolished RIG-I-IRF7 activation and IFN induction, confirming replication-derived dsRNA as the essential immune trigger. Thus, PHEV deploys its N protein to simultaneously sabotage RIG-I sensing and IRF3 effector functions, enabling covert immune evasion critical for neurotropic pathogenesis.

**IMPORTANCE:**

Porcine hemagglutinating encephalomyelitis virus (PHEV) causes lethal encephalomyelitis in piglets by exploiting neuronal immune vulnerabilities. We reveal that PHEV nucleocapsid (N) protein directly binds RIG-I to block its antiviral activation signal (K63-ubiquitination) and concurrently disabling IRF3—the master regulator of early interferon defense. This unique strategy, distinct from nonstructural protein-mediated evasion in other coronaviruses, allows unchecked viral replication during critical early infection. Our work identifies the N protein as a central immunosuppressor evolved for neurotropism and exposes the RIG-I-IRF3 interface as a druggable target. These findings provide a blueprint for countermeasures against PHEV and related neuroinvasive coronaviruses threatening human and animal health.

## INTRODUCTION

The innate immune system is the primary defense against viral infections, relying on pattern recognition receptors (PRRs) like RIG-I to detect viral RNA and initiate interferon (IFN)-mediated antiviral responses (1–3). Activation of RIG-I triggers a signaling cascade via MAVS, leading to phosphorylation and nuclear translocation of transcription factors IRF3 and IRF7, which drive type I IFN production (4, 5). While IRF3 is essential for early IFN induction, IRF7 amplifies late-phase responses, creating a feedforward loop critical for viral control (6–8). Coronaviruses (CoVs), however, frequently evade this system by targeting PRR signaling nodes with viral proteins, enabling persistent infection (9–11).

Porcine hemagglutinating encephalomyelitis virus (PHEV), a neurotropic betacoronavirus, causes fatal encephalomyelitis in piglets (12, 13). Its ability to establish neuronal persistence suggests sophisticated immune evasion (14), yet the mechanisms remain poorly defined. Neurotropic viruses face unique challenges: neurons exhibit dampened innate immunity (e.g., low basal IRF7), and uncontrolled inflammation risks immunopathology (15–17). While CoVs often employ nonstructural proteins (nsps) to suppress IFN, structural proteins like the nucleocapsid (N) protein are emerging as key immunomodulators (16, 18). For instance, SARS-CoV-2 N protein sequesters viral RNA to limit RIG-I sensing (19), and PDCoV N disrupts JAK-STAT signaling (20). However, whether PHEV N protein antagonizes RIG-IRF3/IRF7 pathways—especially in nerve cells—remains elusive.

Roles of IRF3 and IRF7 in virus infection are context-dependent divergence, and their temporal coordination determines the efficacy of host defense. IRF3 typically mediates rapid IFN mRNA transcription, while IRF7, often IFN-inducible, sustains late-phase responses (3, 21). Some neurotropic viruses (e.g., rabies) inhibit IRF3 phosphorylation (22), whereas others (e.g., West Nile virus) exploit delayed IRF7 activation (21). For PHEV, preliminary data suggest blunted IFN responses during early infection (14, 23), but the underlying mechanisms and relative contributions of IRF3/IRF7 are unexplored. Given the dual roles of N protein in CoV replication and immune evasion (19, 20, 24), we hypothesized that PHEV N disrupts RIG-I-dependent signaling to disable IRF3 or IRF7 functions.

Here, we demonstrate that PHEV deploys a two-pronged strategy to evade innate immunity. First, PHEV induced temporal delay in IRF7-dependent IFN-I responses, permitting unchecked viral replication prior to late-phase immunity. Second, PHEV N protein-mediated suppression of RIG-I and IRF3 activation is achieved by direct binding of N to caspase activation and recruitment domain (CARD) of RIG-I to block TRIM25-mediated K63 ubiquitination and downstream IRF3 activation. Our findings unveil PHEV N protein as a crucial molecular switch that concurrently disrupts RIG-I ubiquitination and IRF3 activation—a unique immune evasion mechanism enabling covert immune evasion. These findings redefine N protein multifunctionality in CoV pathogenesis and highlight therapeutic targets for restoring antiviral immunity.

## RESULTS

### PHEV infection activates IFN-I response through RIG-I-IRF7 signaling

**Fig 1:**
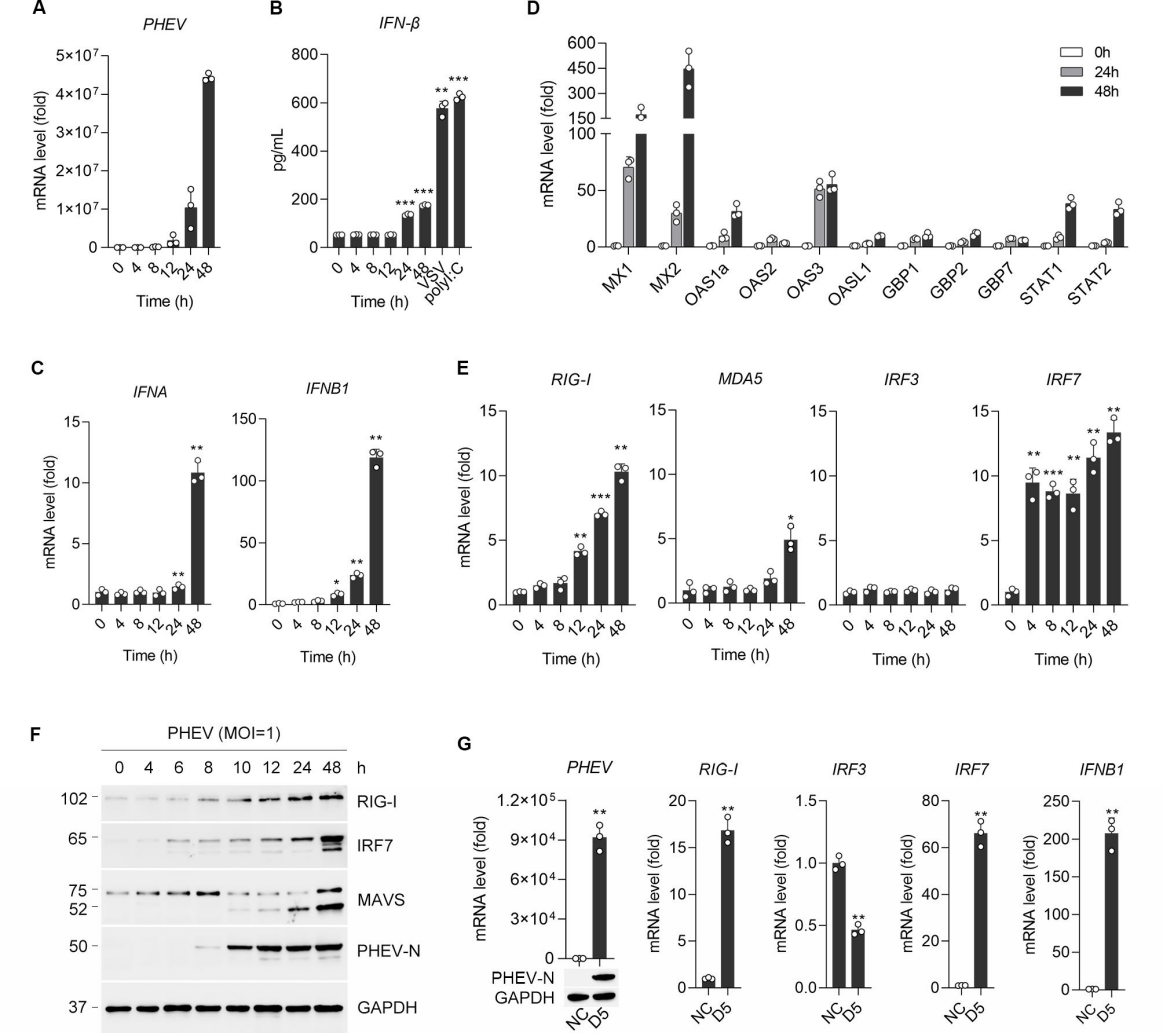
PHEV infection activates the innate immune response. (**A**) Viral load in subcultures harvested at different time points post-infection was quantified by qRT-PCR targeting the viral N gene. All the experiments were performed in triplicate. (**B**) IFN-β levels in PHEV-infected samples were determined by ELISA. Cells infected with VSV (MOI = 1) or treated with Poly(I:C) (20 μM, 24 h) served as positive controls. (**C**) QRT-PCR analysis of IFNA and IFNB1 mRNA expression in N2a cells at various time points (0–48 h) post-PHEV infection. (**D**) QRT-PCR analysis of ISGs (Mx, OAS, GBP, STAT) expression in N2a cells at 24 and 48 h post-PHEV infection. (**E**) QRT-PCR analysis of RIG-I, MDA5, IRF3, and IRF7 expression in N2a cells at various time points (0–48 h) post-PHEV infection. (**F**) WB analysis of RIG-I, IRF7, MAVS, and viral N protein levels in N2a cells at indicated times (0–48 h) after PHEV infection. (**G**) Detection of PHEV, RIG-I, IRF3, IRF7, and IFNB1 in brain tissues from mice at 5 days post-PHEV infection. Data represent mean ± SD (**P < 0.01 and ***P < 0.001 by unpaired two-tailed Student’s t-test).

In PHEV-infected N2a cells, IFN-α/β production and ISGs expression (Mx1, OAS1, GBP1, STAT1) were undetectable before 12 hours post-infection (hpi), coinciding with peak viral replication (Fig. 1A through D). To investigate the underlying mechanism of early-phase IFN-β suppression (<12 hpi), we assessed activation of cytoplasmic RNA sensors RIG-I and MDA5 and downstream signaling. Despite robust activation of the RIG-I-MAVS-IRF7 axis from 12 hpi onward (Fig. 1E and F), this delayed IFN-β response failed to restrict viral propagation (Fig. 1F). Consistently, this effect was also observed in pig kidney cells (PK-15) (Fig. S1). Crucially, intranasal infection of mice with PHEV (10^4.5^ TCID_50_) recapitulated delayed IFN kinetics alongside sustained CNS viral replication (Fig. 1G). These findings establish that PHEV exploits temporally constrained IRF7-dependent signaling to evade early innate immune surveillance in neurons.

### PHEV replication generates the dsRNA required for IFN induction

**Fig 2:**
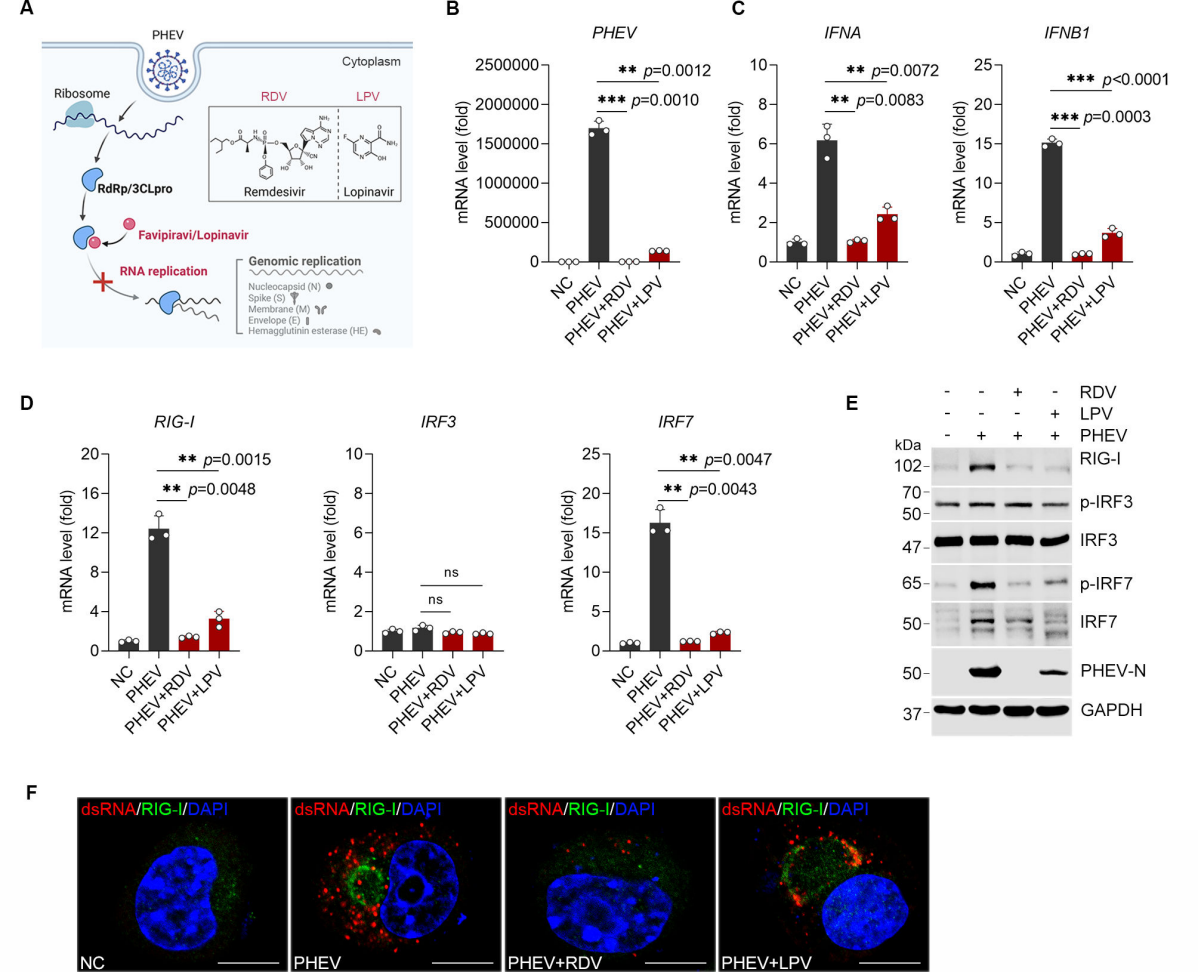
PHEV dsRNA serves as a prerequisite for RIG-I-dependent induction of type I IFN. (**A**) Schematic illustration of the proposed mechanisms by which Remdesivir (RDV) and Lopinavir (LPV) suppress PHEV replication. RdRp, RNA-dependent RNA polymerase; 3CLpro, 3C-like protease. (**B**) RDV and LPV suppress PHEV replication. N2a cells were infected with PHEV for 1 h, treated with RDV or LPV (20 μM), and harvested at 24 h post-infection for qRT-PCR analysis of PHEV mRNA levels. (**C**) QRT-PCR analysis of IFNA and IFNB1 mRNA expression as described in panel **B**. (**D**) QRT-PCR analysis of I RIG-I, IRF3, and IRF7 mRNA expression as described in panel **B**. (**E**) WB analysis of RIG-I, phosphorylated IRF3 (p-IRF3), p-IRF7, and PHEV N protein as described in panel **B**. (**F**) Immunostaining assay. N2a cells as indicated in panel **B** were harvested and immunostained with anti-dsRNA (red) and anti-RIG-I (green) antibodies, DAPI (blue). Scale bar, 10 µm. Data represent mean ± SD (**P < 0.01 and ***P < 0.001 by unpaired two-tailed Student’s t-test).

To establish whether RIG-I-mediated IFN-I induction requires *de novo* viral RNA synthesis, we blocked PHEV replication using Remdesivir (RDV, a viral RNA-dependent RNA polymerase [RdRp] inhibitor) or Lopinavir (LPV, a viral 3-chymotrypsin-like protease [3CLpro] inhibitor) in N2a cells (Fig. 2A). Both inhibitors dramatically suppressed viral genomic RNA replication and abolished subsequent IFN-α/β mRNA expression (Fig. 2B and C). Meanwhile, RDV or LPV treatment would impair the expression of RIG-I and IRF7 mRNA and the activation of the RIG-I-IRF7 signaling axis (Fig. 2D and E). Critically, immunofluorescence revealed that cytoplasmic RIG-I aggregates colocalizing with PHEV dsRNA—indicative of replication-dependent sensing complexes—were abolished by replication inhibitors (Fig. 2F). These data demonstrate that PHEV replication generates immunostimulatory dsRNA, which is indispensable for RIG-I-dependent IFN-I initiation.

### IRF3 is the primary mediator of early antiviral defense against PHEV

**Fig 3:**
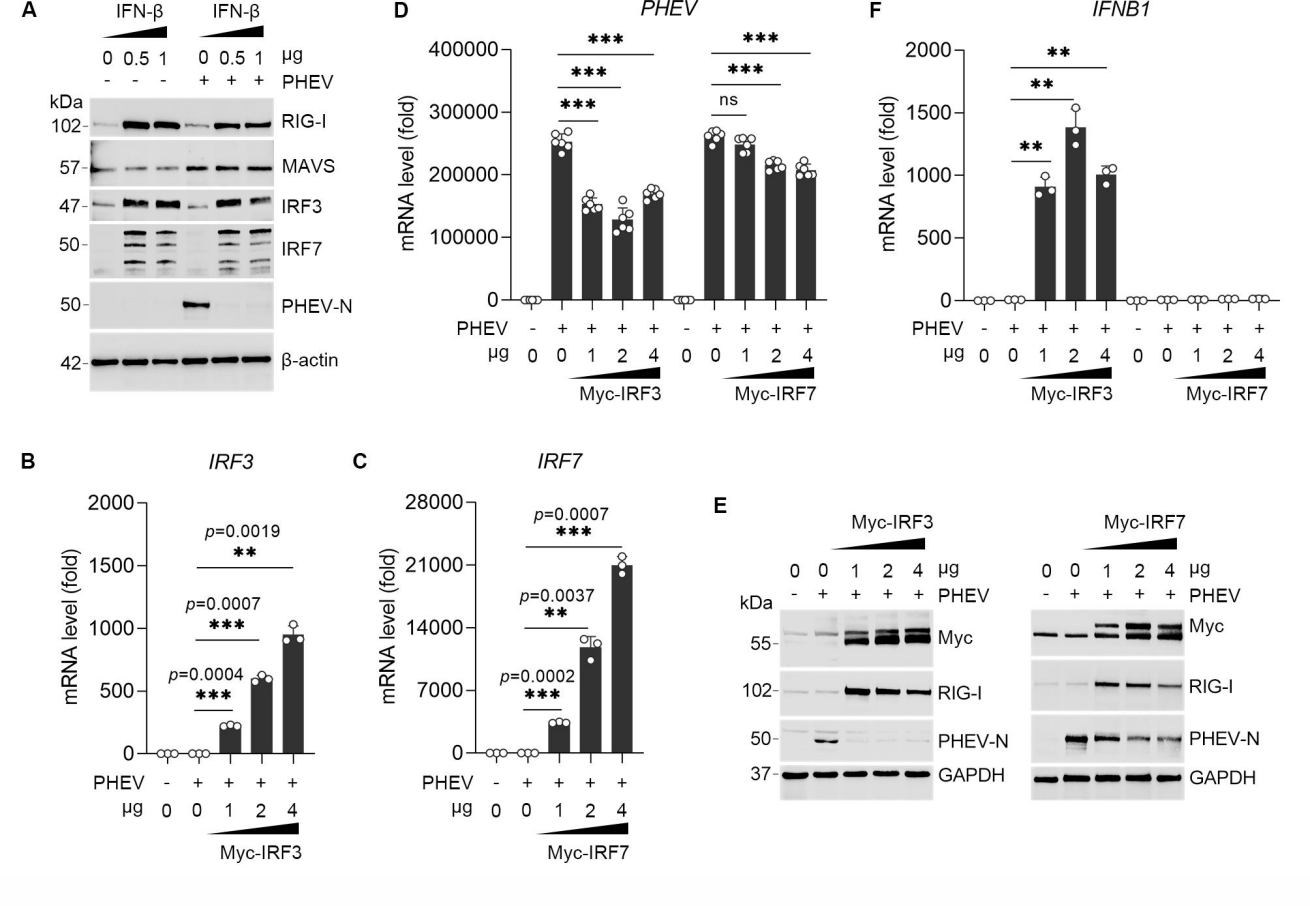
IRF3 initiates the interferon response to suppress viral replication. (**A**) Inhibition of PHEV replication by recombinant IFN-β. WB analysis of viral protein levels to evaluate the suppression of PHEV replication by recombinant IFN-β (0.5 or 1 μg/mL). (**B**) QRT-PCR analysis of IRF3 mRNA in N2a cells overexpressing Myc-tagged IRF3 (1, 2, or 4 μg) for 24 h, followed by 24 h PHEV infection. (**C**) QRT-PCR analysis of IRF7 mRNA in N2a cells overexpressing Myc-tagged IRF7 (1, 2, or 4 μg) for 24 h, followed by 24 h PHEV infection. (**D**) QRT-PCR analysis of PHEV mRNA expression as described in panels **B** and **C**. (**E**) WB analysis of PHEV N protein as described in panels **B** and **C**. (**F**) QRT-PCR analysis of IFNB1 mRNA expression as described in panels **B** and **C**. Data represent mean ± SD (**P < 0.01 and ***P < 0.001 by unpaired two-tailed Student’s t-test). ns, not significant.

Having established that PHEV replication is necessary to trigger RIG-I pathway activation, we next sought to delineate the mechanisms governing the early IFN-β response. Pretreatment with recombinant IFN-β potently suppressed PHEV replication and upregulated IRF3 and IRF7 (Fig. 3A), confirming the virus’s susceptibility to the interferon system. To dissect the respective contributions of the key transcription factors IRF3 and IRF7, we assessed the antiviral effects of their overexpression (Fig. 3B and C). Ectopic expression of IRF3 markedly inhibited PHEV replication, reducing viral mRNA levels by approximately 50% and suppressing viral nucleocapsid (N) protein synthesis (Fig. 3D and E). In contrast, IRF7 overexpression exerted only a marginal inhibitory effect on viral replication, which became detectable only at high expression levels (Fig. 3D and E). Furthermore, IRF3 overexpression triggered a profound upregulation of IFN-β mRNA (by up to 1,000-fold), while IRF7 had minimal impact on IFN-β expression (Fig. 3F). This functional disparity indicates that although PHEV infection subverts the initial activation of IRF3 and preferentially induces IRF7 expression (Fig. 1E), IRF3 remains indispensable for initiating the interferon response. Importantly, IRF7 activation cannot fully compensate for the loss of IRF3 function during the early antiviral defense. Together, these results demonstrate that PHEV strategically disrupts the dominant early antiviral checkpoint orchestrated by IRF3, thereby evading innate immune control and facilitating viral replication.

### PHEV nucleocapsid protein suppresses IFN responses by disrupting IRF3 activation and dimerization

**Fig 4:**
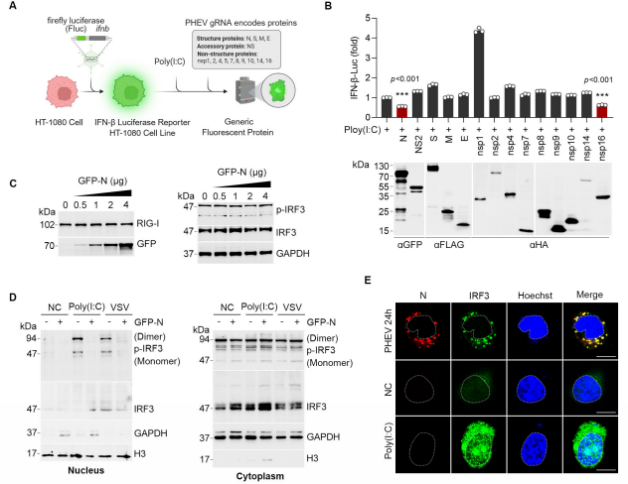
PHEV N protein targets IRF3 to suppress IFN production. (**A**) Schematic illustration of the experimental design for the dual-luciferase assay using HT1080 cells with a stably integrated IFN-β promoter reporter. (**B**) PHEV N protein inhibits the IFN-β promoter. HT1080 cells were transfected with PHEV proteins, cultured for 24 h, and then stimulated with Poly(I:C) (20 μM) for another 24 h to induce IFN-β promoter activity. IFN-β-Luc reporter activity is normalized to that of Renilla luciferase and shown. Detection of viral protein expression by WB. (**C**) WB analysis demonstrated that the protein levels of RIG-I and IRF3 in N2a cells were unaltered by transfection with a gradient of N protein (0.5–4 μg). (**D**) Nuclear-cytoplasmic fractionation. N2a cells were transfected with 2 μg GFP-N recombinant plasmid for 24 h, and then treated with Poly(I:C) (20 μM) for 24 h or infected with VSV (MOI = 1) for 12 h. Cells were collected for cytoplasmic and nuclear isolation. WB analysis of IRF3 nuclear translocation using cytoplasmic and nuclear fractions prepared from harvested cells. (**E**) N2a cells were infected with PHEV for 24 h (with Poly(I:C) treatment (20 μM, 24 h) as a positive control), followed by immunostaining with anti-N (red) and anti-IRF3 (green) antibodies. Nuclei were counterstained with Hoechst (blue). Scale bar, 10 μm. Data represent mean ± SD (**P < 0.01 and ***P < 0.001 by unpaired two-tailed Student’s t-test).

To systematically identify PHEV-encoded proteins capable of antagonizing the RIG-I-IRF3 signaling axis, we performed a functional screening of viral open reading frames (ORFs). This screening utilized a HT-1080 reporter cell line stably harboring an IFN-β promoter-driven firefly luciferase construct. Among all viral proteins tested, the nucleocapsid (N) protein exhibited the most potent suppressive effect, significantly reducing Poly(I:C)-induced IFN-β promoter activation (Fig. 4A and B). This inhibition was not attributable to altered expression of key signaling components, as ectopic expression of the N protein did not affect the endogenous protein levels of RIG-I or IRF3 (Fig. 4C), indicating a specific disruption of signaling transduction rather than protein degradation. We next investigated the stage at which the N protein impedes IRF3-mediated signaling. Nuclear-cytoplasmic fractionation assays demonstrated that N protein abrogated IRF3 homodimerization and nuclear translocation following Poly(I:C) stimulation or VSV infection (Fig. 4D). Corroborating these findings, immunofluorescence analysis demonstrated that while Poly(I:C) treatment robustly induced IRF3 nuclear accumulation, PHEV infection effectively restrained IRF3 in the cytoplasm of N2a cells (Fig. 4E). Collectively, these results establish the PHEV N protein as a direct suppressor that sabotages antiviral immunity through preventing IRF3 homodimerization and subsequent nuclear translocation.

### PHEV N protein targets RIG-I via a CTD-CARD interface to suppress antiviral signaling

**Fig 5:**
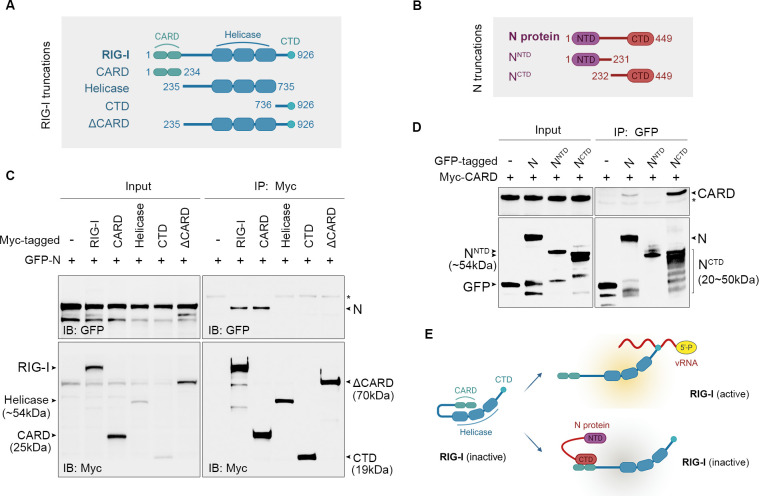
CTD domain of N protein associates with the CARD of RIG-I. (**A**) Schematic structure of RIG-I and the derivatives used in this work. RIG-I (1-926aa), RIG-I-CARD (1-234aa), RIG-I-Helicase (235-735aa), RIG-I-CTD (736-926aa), RIG-I-∆CARD (235-926aa). (**B**) Schematic structure of PHEV N protein and the derivatives used in this work. N(1-449aa), N^NTD^(1-231aa), and N^CTD^(232-449 aa). (**C**) PHEV N protein interacts with the RIG-I CARD domain. HEK293T cells were transfected with the indicated plasmids for 48 h. Cell lysates were immunoprecipitated by using anti-Myc antibody. The precipitates were analyzed by WB using indicated antibodies. (**D**) The CTD of the PHEV N protein interacts with the RIG-I CARD domain. HEK293T cells were transfected with the indicated plasmids for 48 h. Cell lysates were immunoprecipitated by using anti-GFP antibody. The precipitates were analyzed by WB using indicated antibodies. (**E**) Structural schematic of the PHEV N protein-RIG-I interaction. The CTD of PHEV N protein inactivates RIG-I by interacting with the CRAD of RIG-I.

To elucidate the mechanism underlying N protein-mediated suppression of IRF3 activation, we investigated its potential interaction with upstream innate immune sensor RIG-I, which plays a central role in initiating the antiviral signaling cascade. Systematic mapping of the molecular interface was performed using defined truncation constructs of both PHEV N protein (N-terminal domain, N^NTD^; C-terminal domain, N^CTD^) and RIG-I (CARD, Helicase, and CTD domains), as illustrated in Fig. 5A and B. Co-immunoprecipitation assays confirmed a specific physical interaction between full-length N protein and RIG-I, and that this binding requires the C-terminal domain of N (N^CTD^) and the caspase activation and recruitment domain (CARD) of RIG-I (Fig. 5C and D). The critical importance of the RIG-I CARD domain was demonstrated by complete abrogation of binding upon its deletion (ΔCARD; Fig. 5C). These findings mechanistically uncover a direct N^CTD^-CARD interaction between PHEV N protein and innate immune sensor, and this specific molecular engagement disrupts RIG-I’s signaling capacity while preserving its stability, representing a sophisticated strategy for covert immune evasion.

### PHEV N protein competitively blocks RIG-I ubiquitination by disrupting TRIM25 recruitment

**Fig 6:**
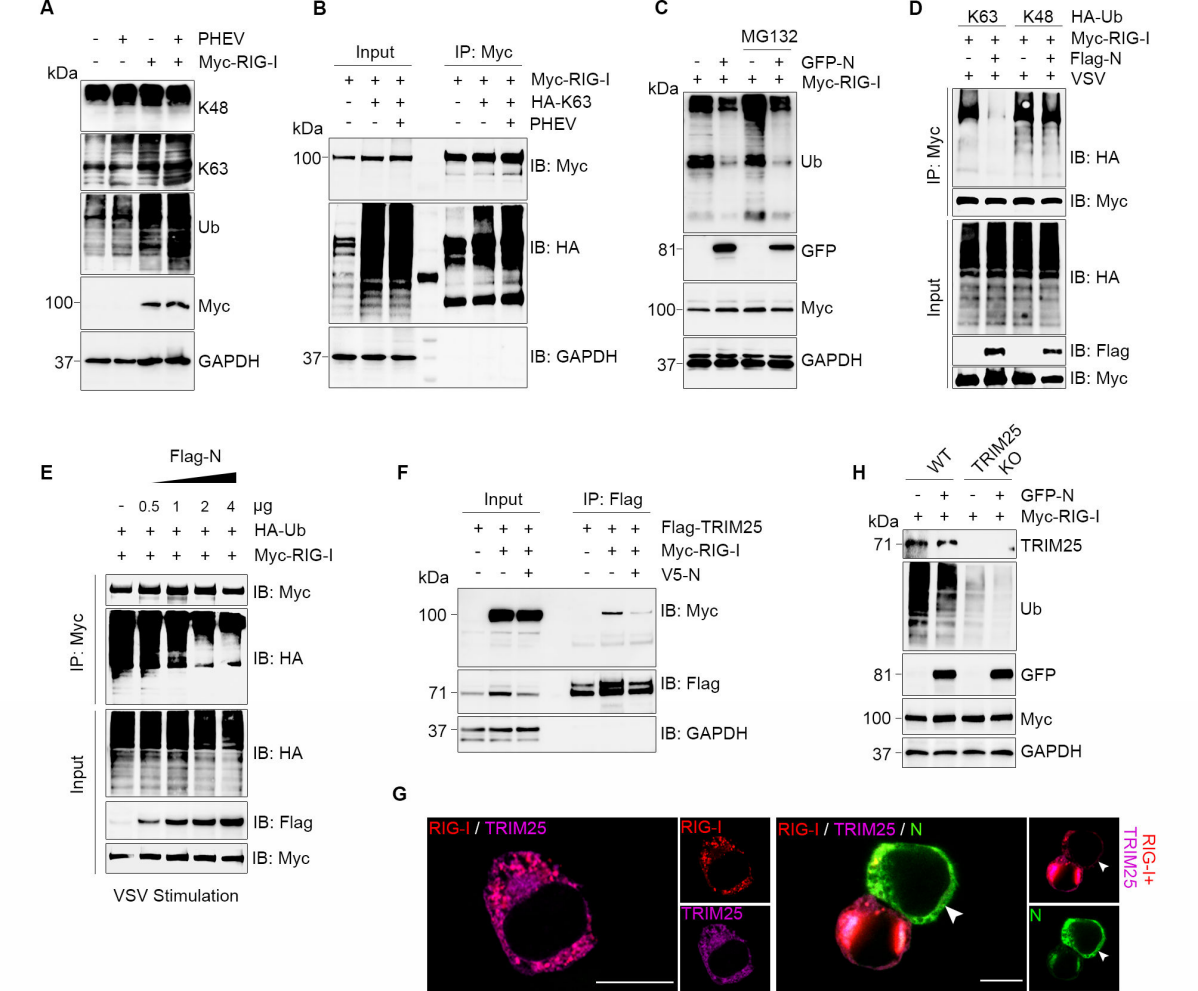
PHEV N protein interrupts the K63-linked polyubiquitination. (**A**) N2a cells were infected with PHEV (MOI = 1) after overexpression of RIG-I for 24 h, and ubiquitination was evaluated by Western blotting. (**B**) N2a cells were co-transfected with Myc-RIG-I and HA-tagged K63 (HA-K63) for 24 h and infected with PHEV for 24 h. Precipitation was performed with Myc antibody and detected by Western blotting. (**C**) N2a cells were transfected with the indicated plasmids for 24 h, and then treated with 20 μM MG132 for 12 h. Cell lysates were collected, and ubiquitination level was detected by Western blotting. (**D**) Effects of N protein on the conjugation of diverse polyubiquitin linkages to RIG-I under viral stimulation. Plasmids encoding HA-Ub (K63, K48), together with expressing vectors for Myc-RIG-I and Flag-N, were co-transfected into HEK293T cells. After 24 h, these cells were infected with VSV for 12 h and then subjected to immunoprecipitation using anti-Myc beads. (**E**) HEK293T cells that were pretransfected with Flag-N-expressing vectors (0.5–4 μg) for 24 h were infected with VSV for 12 h and then subjected to immunoprecipitation using anti-Myc beads. (**F**) HEK293T cells were co-transfected with plasmids encoding Myc-RIG-I, Flag-TRIM25, and V5-N for 48 h. Cell lysates were immunoprecipitated with an anti-Flag antibody and immunoblotted with indicated antibodies. (**G**) Immunostaining assay. White arrows indicate reduced fluorescence signal of RIG-I and TRIM25 co-localization in cells with high N protein expression. Anti-Myc (red), anti-Flag (purple), anti-GFP (green) antibodies, Hoechst (blue). Scale bar, 10 µm. (**H**) Wild type (WT) or TRIM25 knockout (KO) N2a cells were co-transfected with Myc-RIG-I and GFP-N plasmid (2 μg) for 24 h, followed by treatment with MG132 for 12 h. Cell lysates were analyzed by Western blotting.

**Fig 7:**
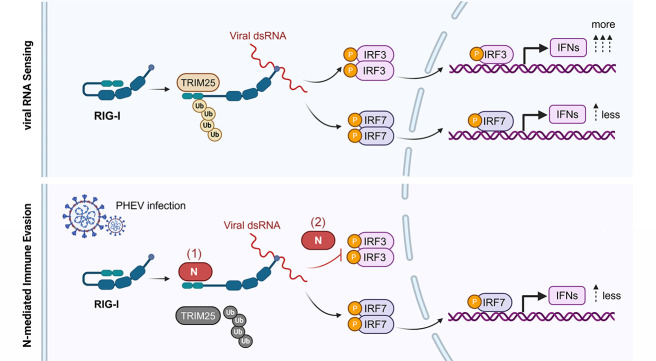
Model of PHEV N protein-mediated immune evasion (1). N protein-mediated competitive inhibition of RIG-I activation: the C-terminal domain (N^CTD^) of the PHEV N protein binds directly to the CARD domain of RIG-I, thereby blocking access of the E3 ligase TRIM25. This prevents TRIM25-mediated K63-linked ubiquitination of RIG-I, which is required for its activation and downstream IFN induction (2). N protein-mediated inhibition of IRF3 phosphorylation. It blocks nuclear translocation of IRF3 and suppresses IRF3-initiated transcription of IFN and ISGs. Consequently, the weak IFN response mediated by IRF7 is insufficient to defend against PHEV infection.

K63-linked polyubiquitination of RIG-I is essential for its activation and subsequent induction of IFN-β. In line with this, we observed that PHEV infection induces K63-linked ubiquitination of RIG-I (Fig. 6A and B). Given that the PHEV N protein interacts with RIG-I and suppresses IFN-β production (Fig. 4 and 5), we next asked whether the N protein impairs RIG-I ubiquitination. Ectopic co-expression of RIG-I and the N protein in N2a cells significantly inhibited RIG-I ubiquitination. This effect was not attributable to proteasomal degradation, as confirmed by MG132 treatment (Fig. 6C). In VSV-stimulated HEK293T cells co-expressing ubiquitin variants (Ub-K63 and Ub-K48) and RIG-I, the N protein specifically reduced K63-linked ubiquitination of RIG-I (Fig. 6D). Furthermore, the N protein inhibited viral stimulation-induced ubiquitination of RIG-I in a dose-dependent manner, without affecting endogenous RIG-I protein levels (Fig. 6E). Mechanistically, the PHEV N protein disrupted the interaction between RIG-I and the E3 ligase TRIM25, reducing TRIM25 binding by approximately 60% (Fig. 6F). Immunofluorescence assay further showed that the N protein inhibits RIG-I/TRIM25 co-localization in cytoplasmic aggregates during infection, consistent with spatial competition (Fig. 6G). Crucially, TRIM25 knockout (KO) in N2a cells substantially diminished the N protein-mediated suppression of RIG-I ubiquitination (Fig. 6H). Collectively, these findings demonstrate that PHEV N protein binding to the CARD domain of RIG-I sterically hinders TRIM25 access, thereby selectively suppressing K63-ubiquitination to silence RIG-I activation while evading immune sensor detection (Fig. 7).

## DISCUSSION

Coronaviruses represent an enduring and multifaceted threat to global health security, with their impact reverberating across medical, economic, and societal domains. Neurotropic coronaviruses, including SARS-CoV-2 (25), human coronavirus OC43 (HCoV-OC43) (26), porcine hemagglutinating encephalomyelitis virus (PHEV) (15), and murine hepatitis virus (MHV) (27) face unique challenges in establishing persistent CNS infections, requiring precise evasion of neuronal innate immunity. Our study delineates a sophisticated dual-layered immune evasion strategy employed by PHEV, wherein the viral N protein concurrently disrupts RIG-I sensing and IRF3 activation to establish early neuronal infection (Fig. 7). This mechanistic divergence from canonical coronavirus IFN antagonism—typically mediated by nonstructural proteins—highlights the N protein evolutionarily as a structural immune modulator tailored for neurotropism.

The repurposing of N protein, a conserved structural protein, for immune antagonism highlights functional plasticity within Betacoronaviruses. Whereas SARS-CoV-2 N protein sequesters viral RNA to limit RIG-I sensing and bind G3BP1 to disrupt stress granules (19, 28), PHEV N protein directly engages CARD of RIG-I, mirroring influenza NS1-mediated RIG-I suppression (29). Its C-terminal domain (CTD) sterically hinders TRIM25 recruitment to RIG-I (Fig. 6), selectively inhibiting K63-linked ubiquitination. Notably, this suppression persisted, though attenuated, in TRIM25-knockout cells, suggesting that the CTD-CARD interaction may also impede alternative E3 ligases (e.g., Riplet) from accessing RIG-I (30). Unlike SARS-CoV-2 PLpro-mediated deubiquitination of RIG-I (18), PDCoV N-mediated STAT1 degradation (20), or coxsackievirus B 3C protease cleaves MAVS (31), PHEV N protein preserves host protein integrity while achieving potent signal suppression. This non-degradative suppression is a stealth strategy minimizing immune sensor (e.g., MDA5, cGAS-STING) alertness, resembling Paramyxovirus V proteins interacting with the RIG-I/TRIM25 regulatory complex (1, 32). In addition, PHEV N protein blocks IRF3 homodimerization, phosphorylation, and nuclear translocation (Fig. 4C through E) without altering total protein levels. The non-degradative suppression of RIG-I and IRF3 by PHEV N protein may represent an evolutionary adaptation to neurotropism. By preserving host protein integrity while inhibiting signaling, the virus minimizes the release of damage-associated molecular patterns and avoids excessive inflammatory cytotoxicity—a critical consideration in post-mitotic neurons where immunopathology can be devastating. This stealth strategy allows sustained viral replication without alerting broader immune surveillance mechanisms, such as MDA5 or cGAS-STING, thereby facilitating persistent CNS infection.

The profound efficacy of exogenous IFN administration in controlling PHEV replication underscores the functional competence of downstream effector mechanisms, highlighting the critical vulnerability specifically at the point of response initiation rather than execution. We demonstrate that PHEV strategically exploits a delayed IRF7-dependent IFN response (<12 h post-infection), which creates a critical permissive window for viral replication. The dominant role of IRF3 in early neuronal defense is consistent with its constitutive expression and rapid activation kinetics, whereas IRF7 fails to compensate during the critical early window. Neurons also exhibit intrinsically low basal IRF7 levels and dampened inflammatory responses, which may further accentuate the reliance on IRF3 for initiating antiviral immunity. Temporally delayed IFN responses also occur in other neurotropic viruses such as West Nile virus (WNV), which compensates for IRF3 inhibition via robust IRF7 amplification (7, 21). PHEV uniquely couples this with targeted IRF3 inactivation. IRF3 dominates early neuronal defense against PHEV (Fig. 3C and D), whereas weak IRF7-driven responses fail to contain early replication. This temporal niche allows unchecked viral propagation prior to late-phase immunity (Fig. 1G), contrasting with SARS-CoV-2 that delays IFN in epithelial cells through distinct mechanisms like Nsp1-mediated translation shutdown and ORF6 blockade of nuclear import for broad-spectrum suppression (33, 34). This nerve-specific vulnerability reflects evolutionary optimization, i.e., disabling the dominant frontline transcription factor (IRF3) while capitalizing on delayed amplifier (IRF7) induction maximizes replication efficiency where inflammatory cytotoxicity must be minimized.

The repurposing of a structural capsid component for immune modulation represents a significant adaptation within Betacoronavirus. While N proteins universally exhibit conserved RNA-binding and nucleocapsid assembly functions (35), PHEV N protein acquires additional specificity for host defense proteins, suggesting convergent evolution in neurotropic viruses. This evolutionary flexibility may explain the success of neurotropic coronaviruses in establishing persistent CNS infections despite constant immune surveillance. Therapeutically, the N-CTD–RIG-I-CARD interface presents a promising target for peptide inhibitors or small molecules to restore RIG-I ubiquitination. Notably, the conservation of this interaction domain in related neurotropic betacoronaviruses (e.g., HCoV-OC43, MHV) warrants exploration of broad-spectrum interventions. Our data further suggest that boosting IRF3 activation, rather than late-phase IRF7 responses, could close the early immune gap exploited by PHEV.

In summary, PHEV orchestrates immune evasion by exploiting a temporal gap in IFN responses and deploying its N protein to disable both RIG-I and IRF3. This dual strategy ensures unchecked viral replication during the critical early phase, enabling CNS invasion and persistence. Our work fundamentally repositions this conserved N protein as a central orchestrator of immune evasion and reveals competitive ubiquitination blockade as a sophisticated stealth tactic evolved for neuronal persistence. These insights establish a comprehensive mechanistic framework for neurotropic coronavirus pathogenesis while illuminating the RIG-I-IRF3 axis as a promising therapeutic target for intervention. It is important to note that while N2a cells provide a useful model for neurotropic virus infection, future studies using primary porcine neuronal cultures will be important to fully validate the physiological relevance of these findings. Future investigations should prioritize several critical directions to extend these findings, i.e., validation in primary porcine neuronal cultures will establish physiological relevance beyond N2a cell lines; comprehensive mapping of TBK1/IKKε-IRF3 phosphorylation dynamics would complement our dimerization and nuclear translocation data to potentially reveal additional regulatory nodes; and cross-species assessment using HCoV-OC43 could uncover conserved evasion principles across betacoronaviruses.

## MATERIALS AND METHODS

### Cells and virus

Mouse neuroblastoma (N2a) (ATCC, CCL-131), PK15 cells (ATCC, CCL-33), and HEK293T (ATCC, CRL-11268) cells were cultured in high-glucose Dulbecco’s modified Eagle’s medium (Gibco, U.S.) with 10% fetal bovine serum (Biological Industries, Israel), and 100 U/mL penicillin, and 100 μg/mL streptomycin. The TRIM25-knockout N2a cell line was generated using CRISPR/Cas9-mediated gene editing. Specifically, a pair of single-guide RNA (sgRNA; sgRNA-F, caccggaacacggtaatgtgcgcgg; sgRNA-R, aaacccgcgcacattaccgtgttcc) targeting exonic regions of the murine TRIM25 gene (NCBI Reference Sequence: NC_000077.7) was cloned into a lentiviral Cas9 vector, and transduced cells were selected by puromycin to establish a clonal knockout cell line. The PHEV strain used in the study was strain CC14 (GenBank: AY048917). Vesicular stomatitis virus (VSV) was produced in N2a cells and stored at −80°C until use.

### Animal experiments

Three-week-old C57 mice (male) were obtained from the Laboratory Animal Center of Jilin University. The mice were intranasally inoculated with 50 µL PHEV (10^4.5^ TCID_50_). Subsequently, PHEV-infected mice at 5 dpi were sacrificed by CO_2_ asphyxiation according to animal handling guidelines. After sacrifice, collected mouse brain tissue for further testing. All animal experiments involving mice were conducted in strict accordance with the Regulations for the Administration of Affairs Concerning Experimental Animals, as approved by the State Council of the People’s Republic of China and the Institutional Animal Care and Use Committee of Jilin University (number of permit: KT202003232). All procedures were designed to minimize animal suffering. Animals were housed in a specific pathogen-free facility with *ad libitum* access to food and water under 22°C ± 1°C, 50% humidity.

### Antibodies and reagent

The following primary antibodies were used: anti-RIG-I antibody (CST, 3743S), anti-MAVs antibody (CST, 4983S), anti-IRF7 antibody (CST, 72073S), anti-IRF3 antibody (CST, 4302S), anti-p-IRF7 antibody (CST, 24129S), anti-p-IRF3 antibody (CST, 79945S), anti-dsRNA antibody (SCICONS, 10,010,500), anti-Ubiquitin (CST, 20326S), anti-TRIM25 (Abcam, ab167154), anti-Myc Monoclonal antibody (Proteintech, 60003-2-Ig), anti-Myc Polyclonal antibody (Proteintech, 16286-1-AP), anti-Flag Monoclonal antibody (Proteintech, 66008-4-Ig), anti-Flag Polyclonal antibody (Proteintech, 20543-1-AP), anti-GFP Monoclonal antibody (Proteintech, 66002-1-Ig), anti-GFP Polyclonal antibody (Proteintech, 50430-2-AP), anti-HA Polyclonal antibody (Proteintech, 51064-2-AP), anti-H3 antibody (Proteintech, 17168-1-AP), and anti-GAPDH antibody (Proteintech, 60,004-1-Ig). The anti-PHEV-nucleocapsid polyclonal antibody was stored in the laboratory. The secondary antibodies used for Western blotting were horseradish peroxidase (HRP)-conjugated anti-mouse or anti-rabbit IgG (Proteintech). The secondary antibodies used for immunofluorescence assay (IFA) were Alexa Fluor 488-conjugated goat anti-mouse/rabbit IgG, Alexa Fluor 594-conjugated goat anti-mouse/rabbit IgG, and Alexa Fluor 647-conjugated goat anti-mouse/rabbit IgG, all of which were purchased from Cell Signaling Technology. Poly(I:C) (HMW) was purchased from invivogen. The Remdesivir and Lopinavir were purchased from MCE. The IFN-β recombinant protein was purchased from MCE. The Antifade Mounting Medium with DAPI and Antifade Mounting Medium with Hoechst 3334 were purchased from Beyotime. The IFN-beta ELISA Kit was purchased from R&D Systems. The Dual-Glo Luciferase Assay System was purchased from Promega. The Lipofectamine 3000, Protein A/G Magnetic Beads, and NE-PER Nuclear and Cytoplasmic Extraction Reagents were purchased from Thermo Fisher.

### Plasmids

Plasmids GFP-N and its N- or C-terminal truncation, V5-N, GFP-NS2, Myc-RIG-I and its truncation forms, Myc-IRF3, Myc-IRF7, HA-Ub, HA-K63, HA-K48, and Flag-TRIM25, were constructed using conventional cloning techniques. The target fragments were amplified by PCR and were then cloned into the pEGFP-C3, pCDNA3.1-V5, pCMV-Myc-N, pCDNA3.1-HA, pCMV-Flag-N vector, respectively. Viral non-structural proteins (nsps) in pCAGGS-HA, Viral structural proteins (S, M, E) in pCAGGS-Flag. All constructs were validated by DNA sequencing.

### Co-immunoprecipitation (Co-IP)

Cells expressing the target proteins were lysed in RIPA buffer (50 mM Tris-HCl pH 7.4, 150 mM NaCl, 1% NP-40, 0.5% sodium deoxycholate, 0.1% SDS) supplemented with protease/phosphatase inhibitors for 30 min on ice. Lysates were centrifuged (12,000 × *g*, 10 min, 4°C) to remove debris and incubated with 1–5 μg of specific antibody (overnight, 4°C). The complex was then incubated with pre-equilibrated protein A/G magnetic beads (2–4 h, 4°C). After washing beads three times with lysis buffer, bound proteins were eluted in 1× loading buffer (62.5 mM Tris-HCl pH 6.8, 2% SDS, 10% glycerol, 0.01% bromophenol blue) by heating at 95°C for 5 min. Eluted proteins were resolved via SDS-PAGE and analyzed by Western blotting. Input lysates (5%–10% of total) were included for validation. All steps were performed at 4°C.

### Dual-luciferase reporter assay

HT1080 cells stably expressing the IFN-β promoter were seeded in 12-well plates and transfected with plasmids encoding PHEV structural or non-structural proteins using Lipofectamine 3000. At 24 h post-transfection, the cells were treated with Poly(I:C) (20 μM, 24 h). Then cells were lysed with Promega Passive Lysis Buffer (PLB), and lysates were assayed sequentially using the Dual-Luciferase Kit: firefly luciferase activity was measured first (Luciferase Assay Reagent II), followed by *Renilla* activity (Stop & Glo Reagent) on a GloMax luminometer. Firefly signals were normalized to *Renilla* (internal control), and data are expressed as fold change relative to controls.

### Nuclear-cytoplasmic fractionation

Nuclear and cytoplasmic fractions were isolated using the Thermo Scientific NE-PER Nuclear and Cytoplasmic Extraction Reagents kit, following the manufacturer’s protocol. Briefly, cultured cells (~80% confluent) were washed with ice-cold PBS, harvested by scraping, and centrifuged at 500 × *g* for 5 min. Cell pellets were resuspended in cytoplasmic extraction reagent (CER I) supplemented with protease/phosphatase inhibitors, vortexed vigorously, and incubated on ice for 10 min. CER II was added, followed by centrifugation at 16,000 × *g* for 5 min at 4°C. The supernatant (cytoplasmic fraction) was collected, and the nuclear pellet was washed with PBS. Nuclear proteins were extracted by resuspending the pellet in nuclear extraction reagent (NER) with protease/phosphatase inhibitors, vortexing intermittently for 40 min on ice, and centrifuging at 16,000 × *g* for 10 min. Protein concentrations of both fractions were quantified via BCA assay. Separation efficiency was validated by Western blotting using Histone H3 (nuclear marker) and GAPDH (cytoplasmic marker).

### Immunofluorescence assay (IFA)

Cultured cells were fixed with 4% paraformaldehyde, permeabilized with Triton X-100 (0.1%–0.5%), and blocked with 5% BSA. Samples were incubated with primary antibodies overnight at 4°C or 1–2 h at room temperature (RT), washed with PBS-Tween, and treated with fluorophore-conjugated secondary antibodies (e.g., Alexa Fluor) for 1 h at RT. Coverslips were mounted using antifade medium containing Hoechst for nuclear counterstaining. Fluorescence was visualized via confocal or fluorescence microscopy.

### Western blotting (WB)

Cultured cells or tissue samples were lysed in RIPA buffer containing protease inhibitors, and protein concentrations were quantified using a BCA assay. Equal amounts of protein (20-50 μg) were separated by SDS-PAGE (8%–12% gels) and transferred to PVDF membranes. Membranes were blocked with 5% skim milk in TBST for 1 h at RT, followed by incubation with primary antibodies (diluted in blocking buffer) overnight at 4°C or 2 h at RT. After washing with TBST, membranes were incubated with HRP-conjugated secondary antibodies (1:5,000–1:10,000) for 1 h at RT. Protein bands were visualized using enhanced chemiluminescence (ECL) and imaged with a ChemiDoc system.

### Quantitative real-time PCR (qRT-PCR)

Total RNA was extracted from cells or tissues using TRIzol reagent, followed by DNase treatment to eliminate genomic DNA. RNA purity and concentration were measured spectrophotometrically (A260/A280 ratio ≥1.8). cDNA was synthesized from 1 μg RNA using reverse transcriptase and oligo(dT) primers. Gene-specific primers (designed via NCBI Primer-BLAST; Tm = 60°C) were validated for amplification efficiency (90%–110%). qRT-PCR reactions (20 μL) included SYBR Green Master Mix, cDNA template, and primers (0.2–0.5 μM), performed in triplicate on a real-time PCR system (e.g., Bio-Rad CFX96) under standardized conditions: 95°C for 3 min, 40 cycles of 95°C for 10 s, 60°C for 30 s, and a melt curve analysis (65°C–95°C). Relative gene expression was calculated using the 2^−ΔΔCt^ method, normalized to β-actin/GAPDH, and expressed as mean ± SD.

### Enzyme-linked immunosorbent assay (ELISA)

Target protein quantification was performed using the RD Systems ELISA Kit (Catalog MIFNB0) according to the manufacturer’s protocol. Briefly, 96-well plates pre-coated with capture antibodies were equilibrated at RT. Standards (recombinant protein serial dilutions) and samples were loaded in duplicate and incubated for 2 h at RT. Plates were washed five times with the provided wash buffer (0.05% Tween-20 in PBS), followed by addition of biotinylated detection antibody (1 h at RT). After washing steps, streptavidin-HRP conjugate was added for 20 min. TMB (3,3′, 5,5″-tetramethylbenzidine) substrate was added to each well and the plate was incubated in the dark at RT for 15 min. Absorbance was measured at 450 nm (reference 570 nm) using a microplate reader. Protein concentrations were calculated using a four-parameter logistic (4-PL) standard curve.

### Statistical analysis

All statistical analyses were performed using GraphPad Prism 8.0 (GraphPad Software, La Jolla, CA, USA). Continuous variables were expressed as mean ± standard deviation (SD). Statistical significance was considered at *P < 0.05, **P < 0.01, and ***P < 0.001.

## Data Availability

The original data obtained in the study are included in the article. Further inquiries can be directed to the corresponding authors.

## SUPPLEMENTAL MATERIAL

Fig. S1 jvi.02112-25-s0001.docx Detection of RIG-I-IRF7 signaling in PHEV-infected PK-15 cells.

ASM does not own the copyrights to Supplemental Material that may be linked to, or accessed through, an article. The authors have granted ASM a non-exclusive, world-wide license to publish the Supplemental Material files. Please contact the corresponding author directly for reuse.
