# Supplementary material for: Porcine hemagglutinating encephalomyelitis virus nucleocapsid protein targets RIG-I and IRF3 to evade IFN immunity
Source: J Virol. 2026 Mar 30;100(4):e02112-25. doi: 10.1128/jvi.02112-25 (PMC13098281; doi:10.1128/jvi.02112-25)
Supplement: Fig. S1 — Detection of RIG-I-IRF7 signaling in PHEV-infected PK-15 cells. [file jvi.02112-25-s0001.docx]

**Supplemental Material**

**Porcine hemagglutinating encephalomyelitis virus nucleocapsid protein targets RIG-I and IRF3 to evade IFN immunity**

Shaoqian Mu^1¶^, Yuanmao Bai^1¶^, Ruizhao Qiu^1^, Feilin Zhang^1^, Junchao Shi^1^, Yungang Lan^1^, Feng Gao^1^, Wenqi He^1*^, Zi Li^1*^

^1^ State Key Laboratory for Diagnosis and Treatment of Severe Zoonotic Infectious Diseases, Key Laboratory for Zoonosis Research of the Ministry of Education, Institute of Zoonosis, and College of Veterinary Medicine, Jilin University, Changchun 130062, China

*** Correspondence:**

Zi Li, e-mail: [lizi@jlu.edu.cn](mailto:lizi@jlu.edu.cn;)

Wenqi He, e-mail: [hewq@jlu.edu.cn](mailto:lizi@jlu.edu.cn;)

^¶^ These authors contributed equally to this work.

**
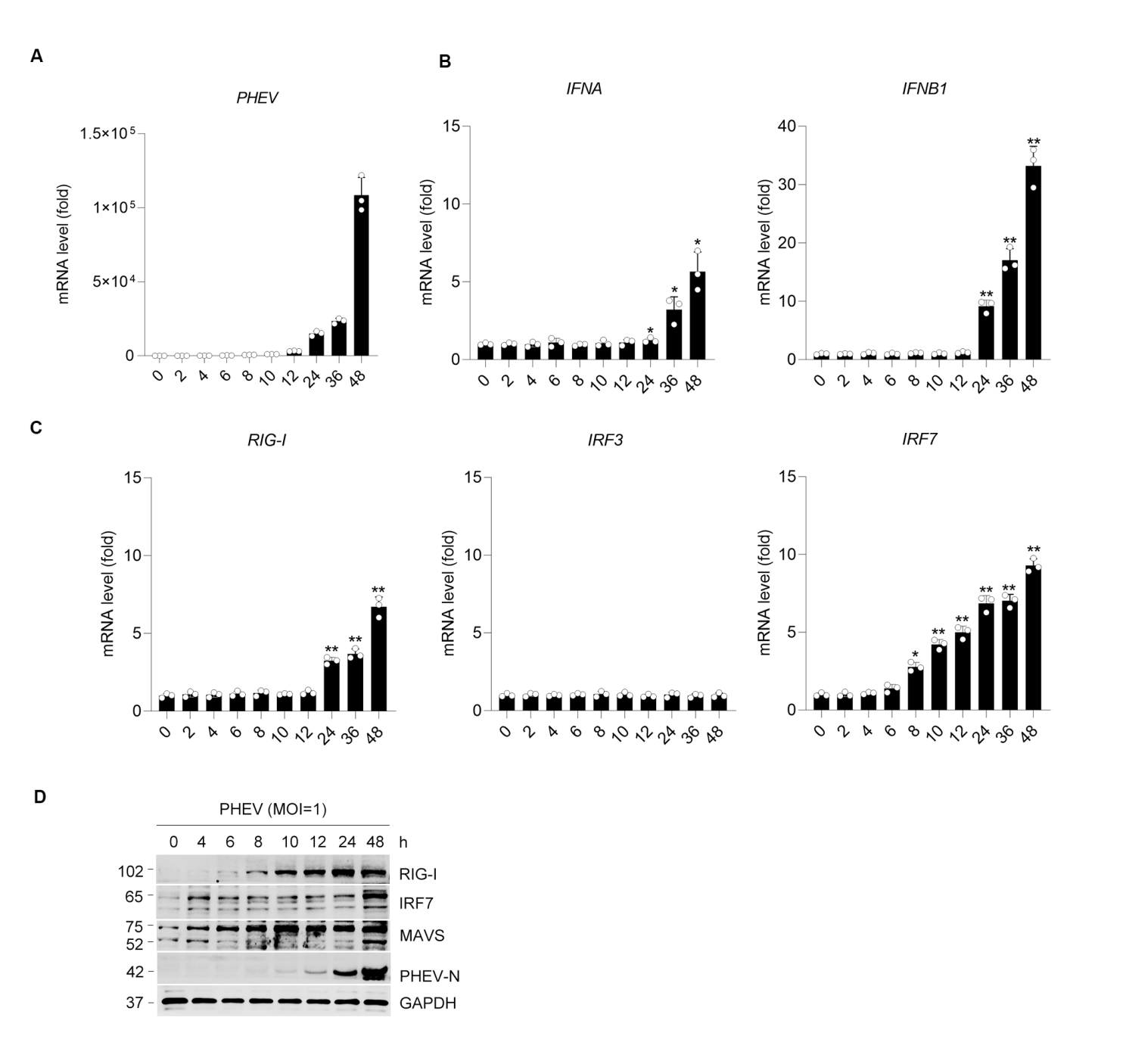
**

**Fig S1. Detection of RIG-I-IRF7 signaling in PHEV-infected PK-15 cells.**

**(A)** Viral load in subcultures harvested at different time points post-infection was quantified by qRT-PCR targeting the viral N gene. All the experiments were performed in triplicate. **(B)** QRT-PCR analysis of IFNA and IFNB1 mRNA expression in PK-15 cells at various time points (0-48 h) post-PHEV infection. **(C)** QRT-PCR analysis of RIG-I, IRF3, and IRF7 expression in PK-15 cells at various time points (0-48 h) post-PHEV infection. **(D)** WB analysis of RIG-I, IRF7, MAVS, and viral N protein levels in PK-15 cells at indicated times (0–48 h) after PHEV infection. Data represent mean ± SD (**P < 0.01 and ***P < 0.001 by unpaired two-tailed Student’s t test).
